# Nrf2 deficiency aggravates PM_2.5_-induced cardiomyopathy by enhancing oxidative stress, fibrosis and inflammation via RIPK3-regulated mitochondrial disorder

**DOI:** 10.18632/aging.102906

**Published:** 2020-03-17

**Authors:** Chenxu Ge, Linfeng Hu, Deshuai Lou, Qiang Li, Jing Feng, Yekuan Wu, Jun Tan, Minxuan Xu

**Affiliations:** 1 Chongqing Key Laboratory of Medicinal Resources in the Three Gorges Reservoir Region, School of Biological and Chemical Engineering, Chongqing University of Education, Chongqing 400067, PR China; 2 Key Laboratory of Biorheological Science and Technology, Ministry of Education, College of Bioengineering, Chongqing University, Chongqing 400030, China; 3 Research Center of Brain Intellectual Promotion and Development for Children Aged 0-6 Years, Chongqing University of Education, Chongqing 400067, PR China

**Keywords:** PM _2.5_, cardiomyopathy, Nrf2, RIPK3, mitochondrial dysfunction

## Abstract

PM_2.5_ is a well-known air pollutant threatening public health, and long-term exposure to PM_2.5_ increases the risk of cardiovascular diseases. Nrf2 plays a pivotal role in the amelioration of PM_2.5_-induced lung injury. However, if Nrf2 is involved in PM_2.5_-induced heart injury, and the underlying molecular mechanisms have not been explored. In this study, wild type (Nrf2^+/+^) and Nrf2 knockout (Nrf2^-/-^) mice were exposed to PM_2.5_ for 6 months. After PM_2.5_ exposure, Nrf2^-/-^ mice developed severe physiological changes, lung injury and cardiac dysfunction. In the PM_2.5_-exposed hearts, Nrf2 deficiency caused significant collagen accumulation through promoting the expression of fibrosis-associated signals. Additionally, Nrf2^-/-^ mice exhibited greater oxidative stress in cardiac tissues after PM_2.5_ exposure. Furthermore, PM_2.5_-induced inflammation in heart samples were accelerated in Nrf2^-/-^ mice through promoting inhibitor of α/nuclear factor κB (IκBα/NF-κB) signaling pathways. We also found that Nrf2^-/-^ aggravated autophagy initiation and glucose metabolism disorder in hearts of mice with PM_2.5_ challenge. Cardiac receptor-interacting protein kinase 3 (RIPK3) expression triggered by PM_2.5_ was further enhanced in mice with the loss of Nrf2. Collectively, these results suggested that strategies for enhancing Nrf2 could be used to treat PM_2.5_-induced cardiovascular diseases.

## INTRODUCTION

Fine particulate matter 2.5 (PM_2.5_) is a collective term referring to atmospheric fine particles, which is regarded as one of the most essential air pollutants in many cities of China [1, 2]. Epidemiological studies have reported that long term exposure to high concentrations of airborne fine PM_2.5_ increases the risk of respiratory and cardiovascular diseases, and animal experiments have showed that PM_2.5_ could induce cardiovascular dysregulation [3–5]. Accumulating evidences demonstrate that the production of ROS, inflammatory responses, calcium homeostasis imbalance and fibrosis are involved in PM-associated cardiovascular disease [6–8]. Nevertheless, there has been only limited data on the detailed molecular mechanisms of PM_2.5_-induced cardiac injury.

Nuclear factor erythroid 2-related factor 2 (Nrf2), a redox-sensitive transcription factor, promotes a battery of antioxidant genes and cytoprotective enzymes that constitute the defense against oxidative stress [9]. Nrf2 activation could protect the heart against ischemia reperfusion injury and diabetic cardiomyopathy [10, 11]. Under normal conditions, Nrf2 is bound to Kelch-like ECH-associated protein 1 (Keap1) in the cytoplasm. However, upon exposure to stressors or inducers, the release of Nrf2 from Keap1 translocates into the nuclear to promote the expression of multiple cytoprotective genes, such as HO1, NAD(P)H: quinone oxidoreductase 1 (NQO1) and glutamate-cysteine ligase modifier (GCLM) [12, 13]. In addition, Nrf2 could modulate the expression of numerous anti-inflammatory and pro-fibrotic genes by antioxidant response elements in their promoters to neutralize free radicals and enhance removal of environmental toxins [14]. Our previous studies indicated that PM_2.5_-induced hypothalamus inflammation and renal injury were associated with the deregulation of Nrf2 signaling, which subsequently influenced inflammatory response both *in vivo* and *in vitro* [15, 16]. In addition, prolonged PM_2.5_ exposure elevates risk of oxidative stress-driven nonalcoholic fatty liver disease partly through the irregular modulation of Nrf2 [17]. Recently, therapeutic strategy to induce Nrf2 expression was effective for the prevention of PM_2.5_-induced lung injury [18]. Considering the critical role of Nrf2 in regulating cardiovascular disease and PM_2.5_-induced tissue injuries, we hypothesized that Nrf2 might also be involved in PM_2.5_-induced heart dysfunction and injury.

The mitochondrion is a sensitive target of both oxidative stress and environmental toxicants stimulus like PM_2.5_ [19, 20]. The abnormal condition of mitochondrial fission and fusion may lead to the irregular alterations of mitochondrial structure and function, which could contribute to respiratory diseases [21]. PM_2.5_ may result in mitochondrial injury in exposed individuals, which in turn at least partly modulates PM-induced cardiovascular injury [22]. The receptor-interacting protein kinase-3 (RIPK3) is a cardinal regulator of necroptosis, and has recently been involved in the pathogenesis of human disease [23, 24]. Recent studies have indicated the increased expression of RIPK3 in murine models of cardiac ischemia/reperfusion injury [25]. We also found that suppressing RIPK3 could alleviate high fat diet-induced hepatic injury partially through the regulation of Nrf2 signaling [26]. Recently, RIPK3 was reported to promote sepsis-triggered acute kidney injury by enhancing mitochondrial dysfunction [27]. Along with the well-documented role of RIPK3-mediated mitophagy in tissue injury, we asked if RIPK3-regulated mitochondrial function could be regulated by Nrf2 in the setting of PM_2.5_-induced cardiomyopathy.

In this study, the wild type (Nrf2^+/+^) and Nrf2 knockout (Nrf2^-/-^) mice were exposed to either ambient PM_2.5_ or filtered air (FA) for 6 months, and then the oxidative stress, fibrosis, inflammation, autophagy, glucose metabolism, RIPK3 expression and mitochondrial function in the hearts were investigated.

## RESULTS

### Effects of Nrf2 deficiency on physiological changes, lung and heart injuries in PM_2.5_-exposed mice

**Figure 1:**
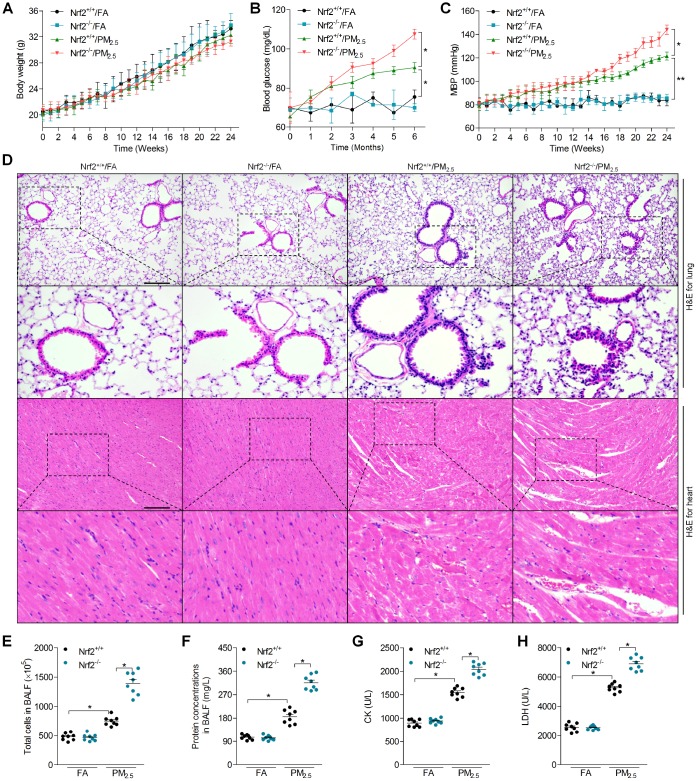
**Effects of Nrf2 deficiency on physiological changes, lung and heart injuries in PM_2.5_-exposed mice.** (**A**) The change of body weight of mice during treatment. n = 15 in each group. (**B**) Calculation of blood glucose. n = 15 in each group. (**C**) MBP of mice from week 1 to week 24. n = 15 in each group. (**D**) H&E staining of lung tissue (up panel) and heart tissue (down panel) sections. n = 6 in each group. Scale bar was 200 μm for the images in the up panels of lung and heart. (**E**) Total cell number and (**F**) protein concentrations in BALF were measured. n = 8 in each group. (**G**) Serum CK and (**H**) LDH levels were determined. n = 8 in each group. Data were expressed as the mean ± SEM. ^*^P < 0.05 and ^**^P < 0.01.

In order to investigate the effects of Nrf2 on PM_2.5_-induced cardiomyopathy, Nrf2^+/+^ and Nrf2^-/-^ mice were used in our present study. Nrf2 was hardly detected in heart and lung tissue samples of Nrf2^-/-^ mice (Supplementary Figure 1A). As shown in Figure 1A, no significant difference was observed in the change of body weight between the Nrf2^+/+^/FA and Nrf2^+/+^/PM_2.5_ groups, or the Nrf2^+/+^/PM_2.5_ and Nrf2^-/-^/PM_2.5_ groups. Significant enhancements of blood glucose were observed in Nrf2^+/+^/PM_2.5_ mice compared to Nrf2^+/+^/FA group. Higher blood glucose levels were detected in Nrf2^-/-^/PM_2.5_ group of mice than that in the Nrf2^+/+^/PM_2.5_ group (Figure 1B). Long term exposure of PM_2.5_ led to significant increases in the mean blood pressure (MBP) of Nrf2^+/+^ mice compared with Nrf2^+/+^/FA mice, which was further accelerated in PM_2.5_-exposed mice with Nrf2^-/-^ (Figure 1C). Subsequently, H&E staining revealed that Nrf2^-/-^ lungs and hearts developed significantly more severe injury than Nrf2^+/+^ lungs in response to PM_2.5_ (Figure 1D). Moreover, PM_2.5_ exposure led to markedly more levels of total cell and higher protein concentration in bronchoalveolar lavage fluid (BALF) from Nrf2^-/-^ mice compared with that from Nrf2^+/+^ mice (Figure 1E and 1F). PM_2.5_-exposure resulted in higher serum creatine kinase (CK) and lactate dehydrogenase (LDH) levels; however, these increases were obviously stronger in serum of Nrf2^-/-^ mice (Figure 1G and 1H). Together, Nrf2 deficiency accelerated PM_2.5_-induced physiological changes, pulmonary and cardiac injuries.

### Nrf2 deletion accelerates cardiac dysfunction and fibrosis in PM_2.5_-exposed mice

**Figure 2:**
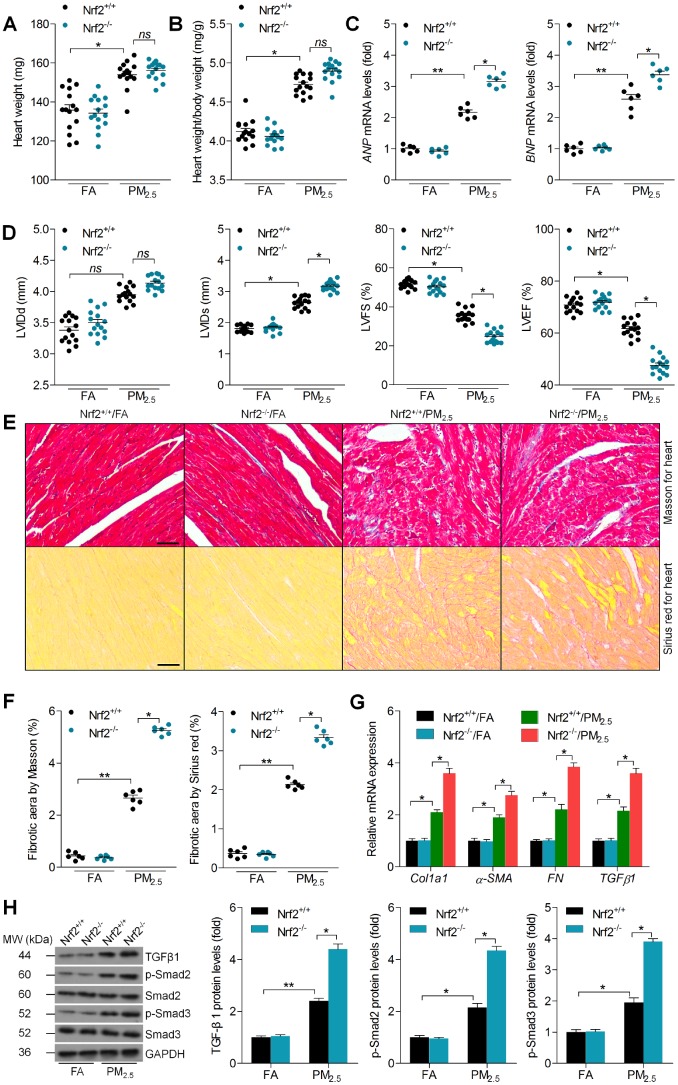
**Nrf2 deletion accelerates cardiac dysfunction and fibrosis in PM_2.5_-exposed mice.** (**A**) Measurements of heart weight. n = 15 in each group. (**B**) Calculation of the ratio of heart weight to body weight. n = 15 in each group. (**C**) RT-qPCR analysis of ANP and BNP mRNA levels in heart samples. n = 6 in each group. (**D**) Cardiac function was analyzed by echocardiography, and LVIDd, LVIDs, LVFS% and LVEF% were quantified. n = 15 in each group. (**E**) Masson’s trichrome staining (up panel) and Sirius Red staining (down panel) of cardiac sections. Scale bar was 100 μm. n = 6 in each group. (**F**) Calculation of fibrotic area following Masson’s trichrome and Sirius Red staining. n = 6 in each group. (**G**) RT-qPCR analysis of Col1a1, α-SMA, FN and TGFβ1 mRNA levels in heart samples. n = 6 in each group. (**H**) Western blot analysis of TGFβ1, p-Smad2 and p-Smad3 protein levels in heart tissues. n = 6 in each group. Data were expressed as the mean ± SEM. ^*^P < 0.05 and ^**^P < 0.01; ns, no significant difference.

In this regard, the effects of Nrf2 deletion on PM_2.5_-induced cardiomyopathy were further investigated. As shown in Figure 2A and 2B, long term exposure of PM_2.5_ led to significant increases in the heart weight and the ratio of heart weight to body weight compared to FA mice from Nrf2^+/+^ group. Though Nrf2^-/-^ promoted these increases, no significant difference was detected after PM_2.5_ stimulation in both genotypes (Figure 2A and 2B). The mRNA levels of atrial natriuretic peptide (ANP) and brain natriuretic peptide (BNP), as essential markers for cardiac hypertrophy that indicates heart injury [28], were significantly up-regulated by PM_2.5_ in Nrf2^+/+^ mice, and these effects were further accelerated in Nrf2^-/-^ mice after PM_2.5_ exposure (Figure 2C). Furthermore, echocardiography analysis demonstrated that PM_2.5_-induced cardiac dysfunction was significantly greater in Nrf2^-/-^ mice, as evidenced by the further increased left ventricular internal diameter during diastole (LVIDd) and LV internal diameter during systole (LVIDs), as well as the decreased LV fractional shortening (LVFS%) and LV ejection fraction (LVEF%) (Figure 2D). As shown in Figure 2E and 2F, Nrf2^-/-^ markedly promoted the fibrotic area in cardiac sections from PM_2.5_-exposed mice compared to Nrf2^+/+^ group. Consistently, PM_2.5_-induced increase of Col1a1, α-SMA, FN and TGFβ1 was further promoted in the Nrf2^-/-^ mice by RT-qPCR analysis (Figure 2G). Exposure to PM_2.5_ increased the TGFβ1, p-Smad2 and p-Smad3 protein levels in the hearts of Nrf2^+/+^ mice; however, these changes were significantly greater in Nrf2^-/-^ mice (Figure 2H). Together, these findings suggested that the cardiac dysfunction and fibrosis in the Nrf2^-/-^ mice were more serious than those in the Nrf2^+/+^ mice after PM_2.5_ exposure.

### Nrf2 knockout promotes PM_2.5_-induced oxidative stress in hart tissues

**Figure 3:**
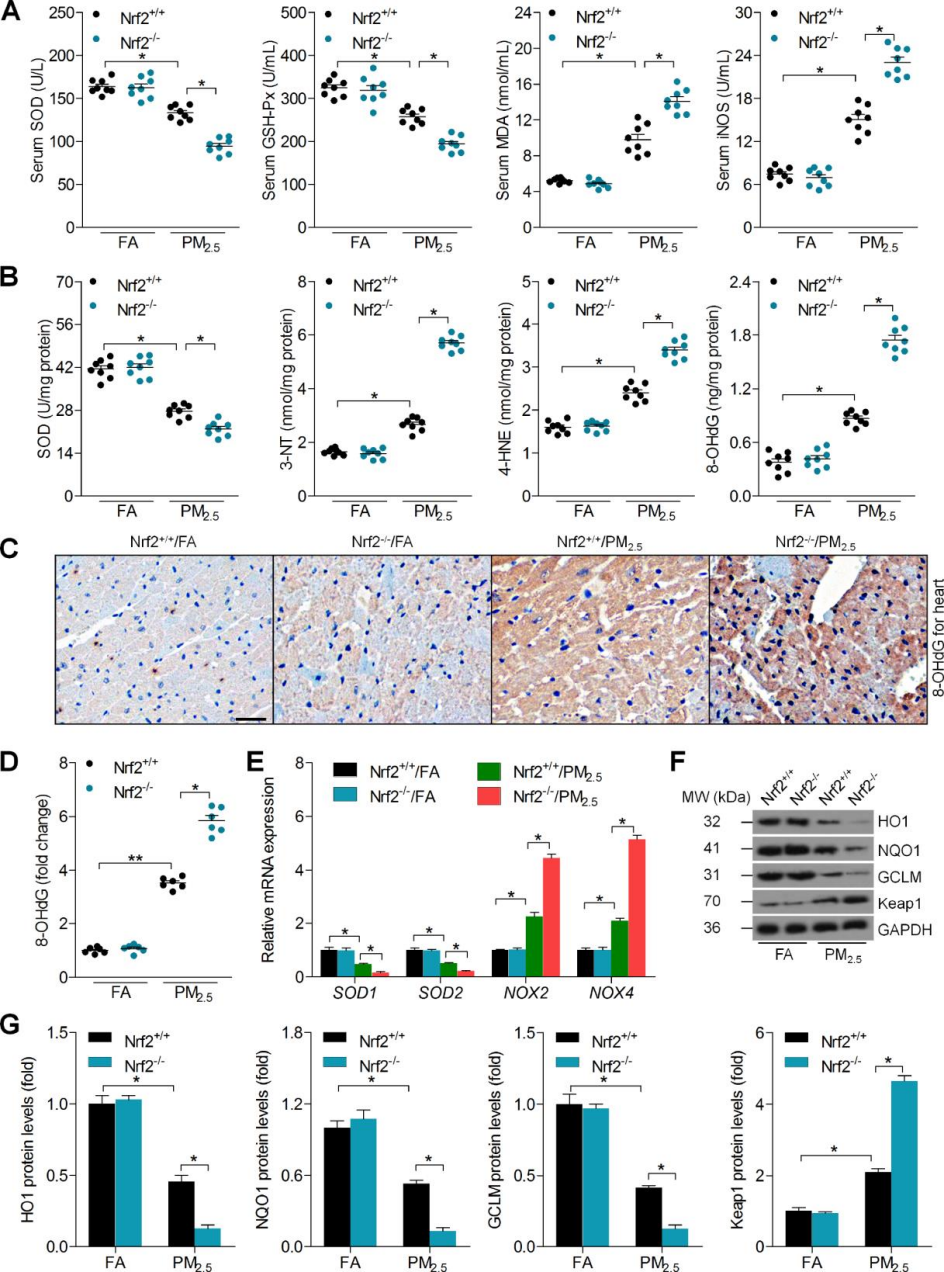
**Nrf2 knockout promotes PM_2.5_-induced oxidative stress in heart tissues.** (**A**) SOD, GSH-Px, MDA and iNOS levels in serum of mice were measured. n = 8 in each group. (**B**) SOD, 3’-NT, 4-HNE and 8-OHdG levels in cardiac samples were determined. n = 8 in each group. (**C**, **D**) IHC staining of 8-OHdG and the quantification of its relative expression were exhibited. Scale bar was 100 μm. n = 6 in each group. (**E**) RT-qPCR analysis of SOD1, SOD2, NOX2 and NOX4 mRNA levels in heart samples. n = 6 in each group. (**F**, **G**) Western blot analysis of HO1, NQO1, GCLM and Keap1 protein expression in heart tissues. n = 6 in each group. Data were expressed as the mean ± SEM. ^*^P < 0.05 and ^**^P < 0.01.

As shown in Figure 3A, long term PM_2.5_ exposure resulted in a significant reduction in serum superoxide dismutase (SOD) and glutathione peroxidase (GSH-Px) of Nrf2^+/+^ mice, which were further decreased in PM_2.5_-induced mice with the loss of Nrf2. However, Nrf2^-/-^ mice developed more malondialdehyde (MDA) and inducible nitric oxide synthase (iNOS) levels in serum compared to Nrf2^+/+^ mice after PM_2.5_ exposure. In addition, Nrf2^-/-^ mice exhibited lower cardiac SOD activity than that of the Nrf2^+/+^ mice exposed to PM_2.5_. Additionally, long term PM_2.5_ exposure led to greater oxidative stress in hearts of Nrf2^-/-^ mice than in hearts of Nrf2^+/+^ mice, as evidenced by higher 3’-Nitrotyrosine (3’-NT), 4-hydroxy-2-nonenal (4-HNE) and 8-hydroxy 2 deoxyguanosine (8-OHdG) levels (Figure 3B). Immunohistochemical (IHC) staining further demonstrated that PM_2.5_-induced 8-OHdG expression in cardiac sections was further accelerated in Nrf2^-/-^ mice (Figure 3C and 3D). Furthermore, Nrf2^-/-^ mice showed lower SOD1 and SOD2 mRNA levels, and higher NOX2 and NOX4 levels than Nrf2^+/+^ mice after PM_2.5_ exposure (Figure 3E). Western blot analysis suggested that the expression of HO1, NQO1 and GCLM was lower, whereas Keap1 expression was higher in Nrf2^-/-^ hearts than in Nrf2^+/+^ hearts (Figure 3F and 3G). Herein, Nrf2 knockout exacerbated PM_2.5_-induced cardiac oxidative stress.

### Nrf2 deficiency enhances cardiac inflammation, RIPK3 expression and mitochondrial disorder in PM_2.5_-exposed mice

**Figure 4:**
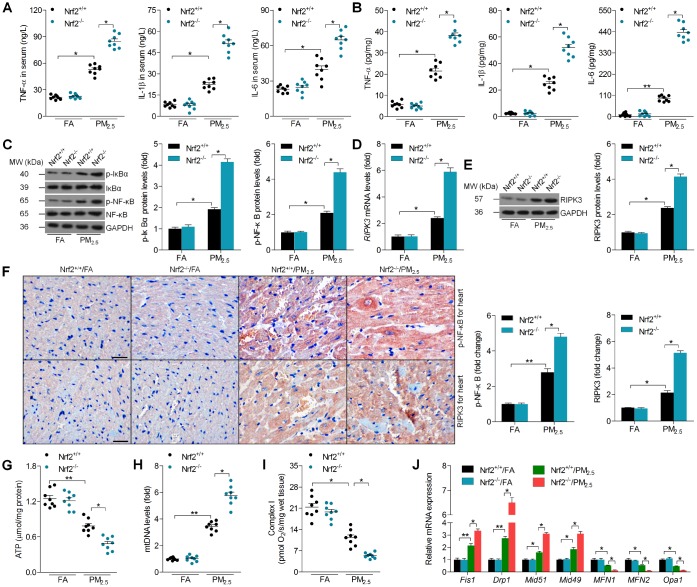
**Nrf2 deficiency enhances cardiac inflammation, RIPK3 expression and mitochondrial disorder in PM_2.5_-exposed mice.** ELISA analysis of TNF-α, IL-1β and IL-6 in (**A**) serum and (**B**) heart tissue samples. n = 8 in each group. (**C**) Western blot analysis of p-IκBα and p-NF-κB in heart tissues. n = 6 in each group. (**D**) RT-qPCR and (**E**) western blot analysis of RIPK3 in heart tissues from each group of mice. n = 6 in each group. (**F**) Representative images for IHC staining of p-NF-κB and RIPK3 in the cardiac sections, and the quantified results of p-NF-κB and RIPK3 were showed. Scale bar was 100 μm. n = 6 in each group. (**G**) ATP production in hearts. n = 8 in each group. (**H**) Content of mtDNA was calculated through the ratio of Cox1 to cyclophilin A. n = 8 in each group. (**I**) Evaluation of complex I respiration rate. n = 8 in each group. (**J**) RT-qPCR analysis of mitochondrial function-associated genes, including Fis1, Drp1, Mid51, Mid49, MFN1, MFN2 and Opa1, in heart tissues. n = 6 in each group. Data were expressed as the mean ± SEM. ^*^P < 0.05 and ^**^P < 0.01.

ELISA analysis indicated that the serum and heart concentrations of TNF-α, IL-1β and IL-6 in Nrf2^-/-^ mice were significantly higher than those in the Nrf2^+/+^ mice after PM_2.5_ exposure (Figure 4A and 4B). We next found that Nrf2^-/-^ mice developed higher expression of p-IκBα and p-NF-κB in hearts compared to the Nrf2^+/+^ mice in response to PM_2.5_ (Figure 4C). According to previous studies, RIPK3 plays a significant role in regulating oxidative stress, fibrosis and inflammation in various types of tissues under different stimuli [23–27]. Subsequently, we found that cardiac RIPK3 expression was markedly up-regulated by PM_2.5_ exposure and was further elevated in the Nrf2^-/-^ mice by RT-qPCR and western blot analysis (Figure 4D and 4E). IHC staining demonstrated that the Nrf2^-/-^ mice exhibited higher expression of RIPK3 and p-NF-κB in cardiac tissue sections than the Nrf2^+/+^ mice after 24 weeks under PM_2.5_ exposure (Figure 4F). Mitochondrial dysfunction shows essential role in meditating ROS production, fibrotic response and inflammatory response during the progression of cardiomyopathy [22, 29, 30]. Then, as displayed in Figure 4G–4I, PM_2.5_ resulted in significant decreases in adenosine triphosphate (ATP) and cardiac fiber OXPHOS capacity (respiration rate at saturating levels of ADP) with complex I-linked substrates, which were further down-regulated in Nrf2^-/-^ mice. In contrast, increased mtDNA levels, a marker of mitochondrial injury, were observed in the hearts of Nrf2^+/+^ mice after PM_2.5_ exposure, and this effect was greater in Nrf2^-/-^ mice with exposure to PM_2.5_. Subsequently, mitochondrial dysfunction-associated genes were measured by RT-qPCR analysis. Nrf2^-/-^ mice showed higher levels of Fis1, Drp1, Mid51 and Mid49 in cardiac samples compared to those from Nrf2^+/+^ mice after PM_2.5_ exposure; however, MFN1, MFN2 and Opa1 mRNA levels reduced by PM_2.5_ were further down-regulated by Nrf2^-/-^ (Figure 4J). Together, the results above demonstrated that Nrf2 deficiency could promote cardiac inflammation, RIPK3 expression and mitochondrial disorder in PM_2.5_-exposed mice.

### Nrf2 loss promotes autophagy initiation and disorder of glucose metabolism in hearts of PM_2.5_-challenged mice

**Figure 5:**
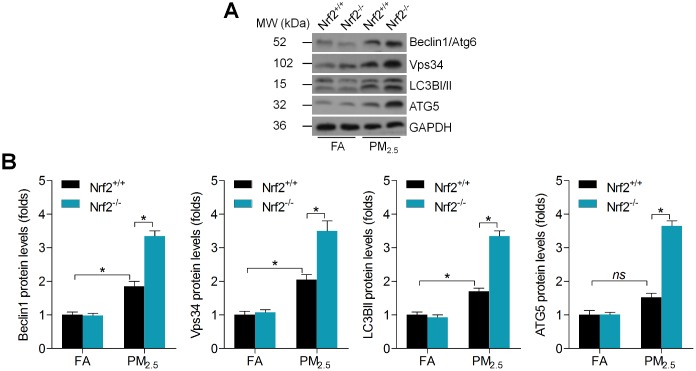
**Nrf2 loss promotes autophagy initiation in PM_2.5_-challenged mice.** (**A**) Representative images of western blotting bands for Beclin1, Vps34, LC3B and ATG5 in cardiac tissues. (**B**) Relative quantification of these molecules by western blot analysis. n = 6 in each group. Data were expressed as the mean ± SEM. ^*^P < 0.05.

Autophagy plays a critical role in regulating the progression of mitochondrial disorder [31]. In addition, PM_2.5_ long-term exposure was reported to induce autophagy in human lung epithelial A549 cells, which was also associated with the induction of oxidative stress [32]. To further explore the molecular mechanisms, we then assessed the protein expression levels of signals involved in autophagy regulation including Beclin1/Atg6, Vps34, LC3B and ATG5. Beclin1/Atg6 forms a complex with Vps34, and this complex is involved in the initiation of autophagy and the formation of autophagosomes [33]. Furthermore, LC3B-II and ATG5 are essential hallmarks of autophagosomes [34]. Western blot results demonstrated that long-term exposure of PM_2.5_ caused higher expression of Beclin1, Vps34, LC3B-II and ATG5 in cardiac tissues of mice, and of note, these effects were markedly accelerated when Nrf2 was knocked out (Figure 5A and 5B). Therefore, we demonstrated that PM_2.5_-induced cardiac injury was associated with the initiation of autophagy.

**Figure 6:**
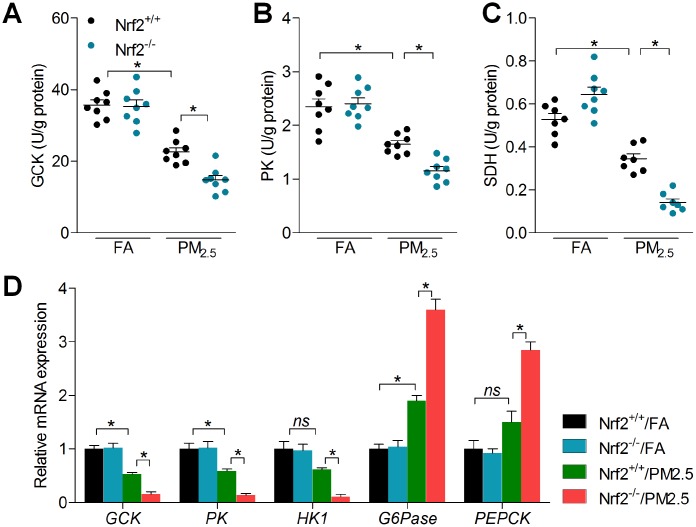
**Nrf2 knockout enhances abnormal glucose metabolism in hearts of PM_2.5_-exposed mice.** (**A**) GCK, (**B**) PK and (**C**) SDH activities in hearts of mice were measured. n = 7 or 8 in each group. (**D**) RT-qPCR was used to measure GCK, PK, HK1, G6Pase and PERCK mRNA levels in hearts of mice. n = 6 in each group. Data were expressed as the mean ± SEM. ^*^P < 0.05; ns, no significant difference.

Moreover, mitochondrial dysfunction was suggested to be involved in the progression of glucose metabolism disorder under different stresses [35, 36]. To further gain insight into the effect of PM_2.5_ on cardiac glucose metabolism, we analyzed the mRNA expression levels of glucose metabolism-related signals. Compared with the FA group, PM_2.5_ treatment led to slight decreases in the expression of GCK, PK, mitochondrial enzyme SDH and hexokinase 1 (HK1) [37–39]; however, gluconeogenesis genes including glucose-6 phosphatase (G6Pase) and phosphoenolpyruvate carboxykinase (PEPCK) were up-regulated by PM_2.5_ [40]. Of note, these effects were markedly exacerbated in mice with Nrf2 deficiency following long-term exposure of PM_2.5_ (Figure 6A–6D). These findings demonstrated that PM_2.5_ challenge mediated gene expression levels of the key rate-limiting enzyme involved in glucose metabolism, which was associated with Nrf2 expression.

### Nrf2-regulated ROS production and RIPK3 expression is involved in PM_2.5_-induced oxidative stress, fibrosis and inflammation *in vitro*

**Figure 7:**
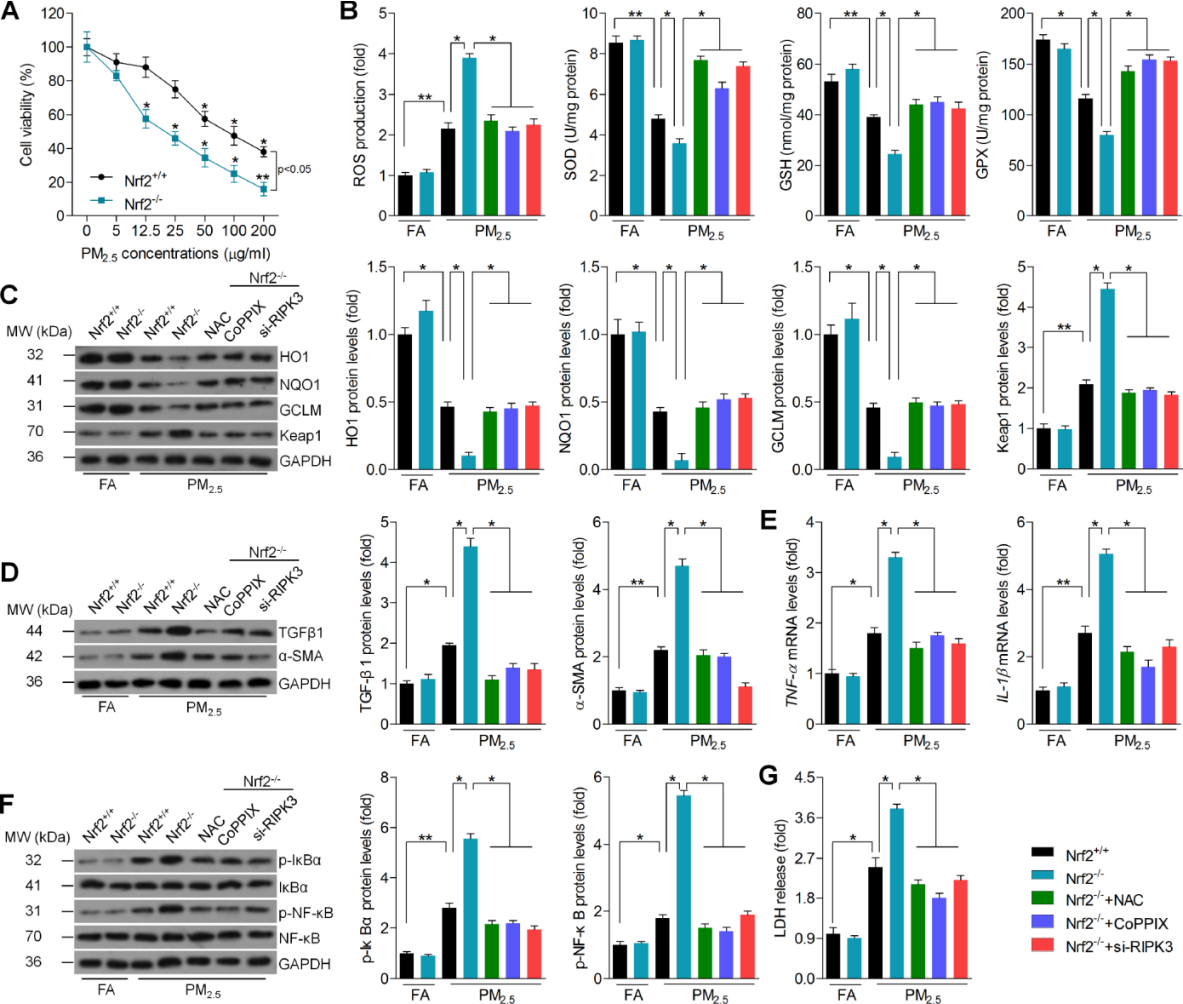
**Nrf2-regulated ROS production and RIPK3 expression is involved in PM_2.5_-induced oxidative stress, fibrosis and inflammation *in vitro*.** (**A**) Cardiomyocytes isolated from Nrf2^+/+^ or Nrf2^-/-^ mice were treated with the indicated concentrations (0, 5, 12.5, 25, 50, 100 and 200 μg/ml) of PM_2.5_ for 24 h. Then, all cells were harvested for cell viability measurement using MTT analysis. n = 8 in each group. (**B**–**G**) Cardiomyocytes isolated from Nrf2^+/+^ or Nrf2^-/-^ mice were pre-treated with NAC (5 mM) or CoPPIX (15 μM) for 2 h, or transfected with si-RIPK3 for 24 h. Then, all cells were incubated with PM_2.5_ (100 μg/ml) for another 24 h. After treatments above, all cells were collected for further calculation. (**B**) Intracellular calculation of ROS production, SOD activity, GSH and GPX levels. n = 8 in each group. (**C**) Western blot analysis of HO1, NQO1, GCLM and Keap1 protein expression in cells. n = 6 in each group. (**D**) Western blot analysis of TGFβ1 and α-SMA in cells. n = 6 in each group. (**E**) RT-qPCR analysis of TNF-α and IL-1β in cells. (**F**) Western blot analysis of p-IκBα and p-NF-κB in cells. n = 6 in each group. (**G**) LDH release in cells. n = 8 in each group. Data were expressed as the mean ± SEM. ^*^P < 0.05 and ^**^P < 0.01.

To further explore the effects of Nrf2 on cardiac injury, *in vitro* experiments were performed. First, the cell viability was investigated using MTT analysis. The results suggested that PM_2.5_ dose-dependently reduced the cell viability of cardiomyocytes isolated from Nrf2^+/+^ mice; however, these effects were further exacerbated in cardiomyocytes isolated from Nrf2^-/-^ mice, demonstrating the pivotal role of Nrf2 in sustaining cell survival rate (Figure 7A). Considering the essential role of oxidative stress and RIPK3 in Nrf2-regulated cardiomyopathy induced by PM_2.5_, ROS scavenger N-acetyl-L-cysteine (NAC), HO1 activator cobalt protoporphyrin IX (CoPPIX) and RIPK3 siRNA (si-RIPK3) were subjected to PM_2.5_-incubated cardiomyocytes isolated from Nrf2^-/-^ mice. The successful transfection efficacy of si-RIPK3 in cardiomyocytes was confirmed by western blot analysis (Supplementary Figure 1B). As shown in Figure 7B, PM_2.5_-induced cellular ROS was further elevated in Nrf2^-/-^ cardiomyocytes. Of note, we found that pre-treatment of NAC, CoPPIX or si-RIPK3 markedly reduced Nrf2^-/-^-elevated ROS generation in PM_2.5_-incubated cardiomyocytes. Inversely, PM_2.5_-decreased concentration of antioxidants, including SOD, glutathione (GSH) and GPX was further reduced by Nrf2^-/-^, and this effect was rescued in response to NAC, CoPPIX or si-RIPK3. HO1, NQO1 and GCLM expression levels were inactivated by PM_2.5_, and were further reduced by Nrf2 knockout. However, addition of NAC, CoPPIX or transfection with si-RIPK3 restored the HO1, NQO1 and GCLM expression despite the Nrf2^-/-^. In contrast, NAC, CoPPIX or si-RIPK3 significantly abrogated Nrf2^-/-^-promoted Keap1 expression in PM_2.5_-incubated cardiomyocytes (Figure 7C). The expression of TGFβ1 and α-SMA was increased in PM_2.5_-treated cells and was further aggravated in response to Nrf2^-/-^. However, blockade of the ROS and RIPK3, or promotion of HO1 significantly abolished the TGFβ1 and α-SMA expression despite Nrf2 deficiency (Figure 7D). Similarly, the inflammatory factors, including TNF-α and IL-1β, were also up-regulated in PM_2.5_-incubated cells, and were accelerated in response to Nrf2 knockout. These effects were significantly nullified by NAC, CoPPIX or si-RIPK3 (Figure 7E). Consistent with the change of TNF-α and IL-1β, similar results were detected in the expression of p-IκBα and p-NF-κB (Figure 7F). Moreover, the content of LDH was increased in PM_2.5_-treated cardiomyocytes, indicative of the breakage of cell membranes due to cell death. Nrf2^-/-^ further enhanced the LDH release, and this effect was reversed by NAC, CoPPIX or si-RIPK3 (Figure 7G). Herein, Nrf2-regulated ROS production and RIPK3 expression was implicated in PM_2.5_-induced oxidative stress, fibrosis and inflammation.

### Nrf2 modulates RIPK3 expression to regulate mitochondrial disorder in PM_2.5_-exposed cardiomyocytes

**Figure 8:**
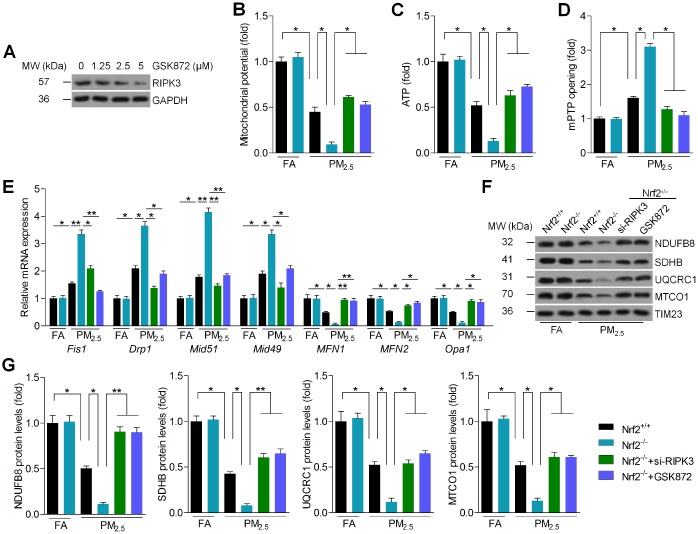
**Nrf2 modulates RIPK3 expression to regulate mitochondrial disorder in PM_2.5_-exposed cardiomyocytes.** (**A**) Cardiomyocytes were treated with the indicated concentrations of GSK872 (0, 1.25, 2.5 and 5 μM) for 2 h, followed by western blot analysis of RIPK3. n = 6 in each group. (**B**–**G**) Cardiomyocytes isolated from Nrf2^+/+^ or Nrf2^-/-^ mice were pre-treated with GSK872 (15 μM) for 2 h, or transfected with si-RIPK3 for 24 h. Then, all cells were exposed to PM_2.5_ (100 μg/ml) for another 24 h. After treatments above, all cells were collected for further studies. (**B**) Mitochondrial potential results by JC-1 analysis. n = 8 in each group. (**C**) Intracellular ATP levels were measured. n = 8 in each group. (**D**) Results of mPTP opening in cells. n = 8 in each group. (**E**) RT-qPCR analysis of Fis1, Drp1, Mid51, Mid49, MFN1, MFN2 and Opa1 in cells. n = 6 in each group. (**F**, **G**) Western blot analysis of NDUFB8, SDHB, UQCRC1 and MTCO1 in the mitochondrial fractions. n = 6 in each group. Data were expressed as the mean ± SEM. ^*^P < 0.05 and ^**^P < 0.01.

RIPK3 has been reported to be involved in the alteration of mitochondrial disorder [27]. Subsequently, RIPK3 expression was further inhibited by addition of GSK872, a RIPK3 inhibitor (Figure 8A). As shown in Figure 8B and 8C, the mitochondrial potential and ATP were significantly reduced by PM_2.5_ treatment. However, RIPK3 knockdown and inhibition by GSK872 rescued the mitochondrial potential and ATP levels in PM_2.5_-treated cardiomyocytes despite Nrf2 knockout. Besides, the opening rate of mitochondrial permeability transition pore (mPTP) was significantly up-regulated in PM_2.5_-treated cells and was further promoted by Nrf2 knockout, which was, however, dramatically decreased by the suppression of RIPK3 (Figure 8D). Then, RT-qPCR analysis suggested that mitochondrial disorder was markedly induced in cardiomyocytes in response to PM_2.5_. However, the loss of Nrf2 promoted PM_2.5_-induced expression of fission protein 1 (Fis1), dynamin-related protein 1 (Drp1), mitochondrial dynamics protein of 51 (Mid51) and mitochondrial dynamics protein of 49 (Mid49), and these effects were considerably blocked by RIPK3 suppression. In contrast, si-RIPK3 or GSK872 pre-treatment significantly restored the expression of mitofusin-1 (MFN1), MFN2 and optic atrophy 1 (Opa1) in PM_2.5_-incubated cardiomyocytes with Nrf2 deficiency when compared to Nrf2^-/-^ cardiomyocytes only treated with PM_2.5_ (Figure 8E). To further determine the effect of RIPK3 on mitochondrial respiratory chain complexes, mitochondrial fractions were isolated from Nrf2^+/+^ and Nrf2^-/-^ cardiomyocytes following PM_2.5_ exposure. Western blots demonstrated that PM_2.5_ treatment significantly reduced the function of mitochondrial complex I, -II -III, and -IV, as evidenced by the reduced expression of NADH dehydrogenase (ubiquinone) 1 beta subcomplex subunit 8 (NDUFB8), succinate dehydrogenase subunit B (SDHB), ubiquinol cytochrome c oxidoreductase core protein (UQCRC1) and mitochondrial cytochrome c oxidase subunit 1 (MTCO1), respectively [41, 42]. These effects were further accelerated by the knockout of Nrf2 in PM_2.5_-incubated cardiomyocytes, but RIPK3 inhibition significantly rescued the expression of these signals (Figure 8F and 8G). Taken together, these results demonstrated that Nrf2^-/-^-enhanced mitochondrial dysfunction could be improved by RIPK3 suppression in PM_2.5_-exposed cardiomyocytes.

### Promoting Nrf2 expression alleviates PM_2.5_-induced cardiomyopathy

**Figure 9:**
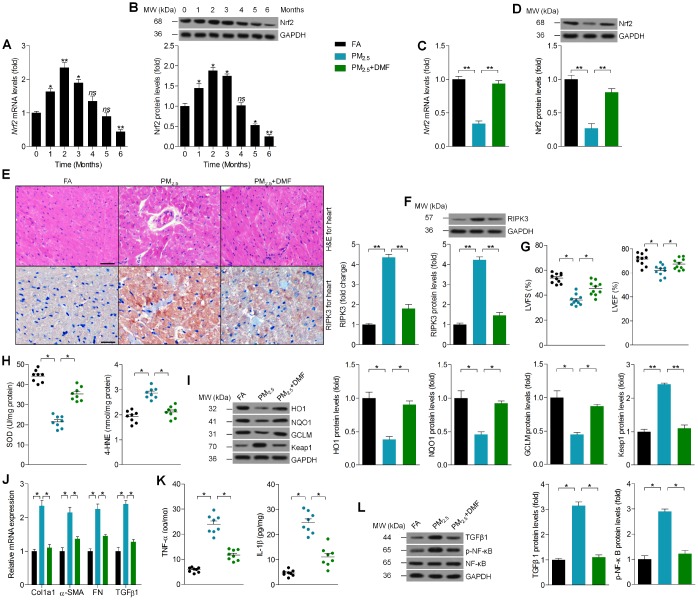
**Promoting Nrf2 expression alleviates PM_2.5_-induced cardiomyopathy.** (**A**) RT-qPCR and (**B**) western blot analysis of Nrf2 in heart tissues from Nrf2^+/+^ mice at the indicated time points. n = 6 in each group. (**C**) RT-qPCR and (**D**) western blot analysis of Nrf2 in heart tissues from Nrf2^+/+^ mice treated with or without DMF. n = 6 in each group. (**E**) Representative images of H&E staining and RIPK3 staining of cardiac sections from the indicated groups of mice were displayed, and the relative expression of RIPK3 was quantified by IHC. n = 6 in each group. (**F**) Western blot analysis of RIPK3 in heart tissues. n = 6 in each group. (**G**) Cardiac function was analyzed by echocardiography. LVFS% and LVEF% were quantified. n = 10 in each group. (**H**) Cardiac SOD and 4-HNE levels were measured. n = 8 in each group. (**I**) Western blot analysis of HO1, NQO1, GCLM and Keap1 protein expression in heart tissues. n = 6 in each group. (**J**) RT-qPCR analysis of Col1a1, α-SMA, FN and TGFβ1 mRNA levels in heart samples. n = 6 in each group. (**K**) ELISA analysis of TNF-α and IL-1β in heart tissues. n = 8 in each group. (**L**) Western blot analysis of TGFβ1 and p-NF-κB in heart tissues. n = 6 in each group. Data were expressed as the mean ± SEM. ^*^P < 0.05 and ^**^P < 0.01; ns, no significant difference.

Finally, RT-qPCR and western blot analysis suggested that Nrf2 mRNA and protein levels in cardiac samples were up-regulated in mice exposed to PM_2.5_ during the beginning two months, and then were down-regulated especially at the end of PM_2.5_ treatment (Figure 9A and 9B). Subsequently, dimethyl fumarate (DMF), as a critical Nrf2 activator [43, 44], was administered to mice with or without PM_2.5_ exposure to further investigate the effects of Nrf2 on PM_2.5_-induced cardiomyopathy. As shown in Figure 9C and 9D, PM_2.5_-reduced expression of Nrf2 in heart tissues was markedly rescued by DMF. H&E staining demonstrated that DMF supplementation improved PM_2.5_-induced change of cardiac histopathology (Figure 9E). In addition, cardiac RIPK3 expression induced by PM_2.5_ was significantly decreased by DMF following IHC and western blot analysis (Figure 9E and 9F). DMF treatment improved PM_2.5_-induced cardiac dysfunction, as evidenced by the increased LVFS% and LVEF% (Figure 9G). Then, significantly up-regulated SOD activity and down-regulated 4-HNE levels were detected in hearts of DMF-treated mice after PM_2.5_ exposure (Figure 9H). As expected, a significant decrease in HO1, NQO1 and GCLM was observed in hearts of PM_2.5_-exposed mice, while Keap1 was increased. These effects were markedly reversed by DMF (Figure 9I). As shown in Figure 9J and 9K, DMF administration dramatically reduced PM_2.5_-induced expression of cardiac Col1a1, α-SMA, FN, TGFβ1, TNF-α and IL-1β compared to PM_2.5_ group. Within expectation, DMF treatment evidently alleviated the expression of TGFβ1 and p-NF-κB in hearts of mice receiving PM_2.5_ (Figure 9L). The *in vivo* results above indicated that promoting Nrf2 expression could alleviate PM_2.5_-induced cardiomyopathy by preventing RIPK3 expression, oxidative stress, fibrosis and inflammation.

## DISCUSSION

Epidemiologic studies have reported that PM_2.5_ pollution is highly associated with cardiovascular diseases, such as stroke, ischemia, arrhythmia and heart failure [1–4]. Nrf2 is a key transcription factor that regulates antioxidant defense [45], and Nrf2 could protect against diverse PM_2.5_ components-induced mitochondrial oxidative damage in lung cells [46]. Here, we demonstrated three major new findings. On the one, our results indicated that long term PM_2.5_ exposure led to more severe cardiac dysfunction and injury in Nrf2^-/-^ mice than that in the Nrf2^+/+^ mice. On the other, the accelerated fibrosis, oxidative stress and inflammation in PM_2.5_-treated Nrf2^-/-^ mice were significantly associated with the abnormal expression of RIPK3 and RIPK3-induced mitochondrial disorder. Finally, we demonstrated that promoting Nrf2 expression by its activator of DMF considerably alleviated PM_2.5_-induced cardiac injury through reducing RIPK3 expression, oxidative stress, fibrosis and inflammatory response. As far as we know, it was the first study that Nrf2 exhibited a significant protective role against PM_2.5_-induced cardiomyopathy through suppressing collagen accumulation, oxidative stress and inflammation regulated by RIPK3, as well as the improvement of autophagy and glucose metabolism in hearts.

Growing studies have indicated that PM_2.5_ contains very complex toxic chemical compositions. The lung tissue is a primary biological target for injury arising from inhalation exposure to PM_2.5_. Airborne pollutants could be released into the pulmonary surfactant and attach to lung epithelial cells, resulting in pulmonary damage [47, 48]. As reported before, therapeutic strategy to induce Nrf2 expression is effective for preventing PM_2.5_-induced lung injury [49]. In our present study, we confirmed that PM_2.5_ exposure led to significant lung injury, along with high cell influx and protein concentration in the extracted BALF, which were however, further accelerated in Nrf2^-/-^ mice after PM_2.5_ exposure. Our previous studies also revealed the critical role of Nrf2 in preventing hypothalamus inflammation, renal injury and liver damage in rodent animals under PM_2.5_ stimuli [15–17]. These findings demonstrated that Nrf2 might be connected to PM_2.5_ particles-induced heart injury. The heart is another important target organ of PM_2.5_ exposure. The direct acute influences of PM_2.5_ on the myocardium involve ROS production, spontaneous arrhythmias and alterations in myocardial blood [50]. In this study, we elucidated that exposure to PM_2.5_ for 6 months led to moderate physiological changes, histopathological changes, collagen accumulation, oxidative stress, inflammatory response and mitochondrial dysfunction in hearts of Nrf2^+/+^ mice. However, these effects were markedly exacerbated by the deficiency of Nrf2. Autophagy is an evolutionarily conserved lysosomal catabolic mechanism involved in degradation of damaged organelles and long-lived proteins in response to unfavorable conditions [31, 33, 34, 51]. *In vivo* evidence showed that excessive autophagy was involved in testicular blood-testis barrier (BTB) damage and toxicity of rats after developmental exposure to the air pollution PM_2.5_, which could be mediated by the increasing ROS [52]. In addition, the PM_2.5_-induced oxidative stress probably plays a key role in autophagy in human lung epithelial A549 cells, which may contribute to PM_2.5_-induced impairment of pulmonary function [53]. In addition, Nrf2 signaling has been demonstrated to control autophagy under various stresses [54, 55]. Similarly, here we found that long-term PM_2.5_ led to the initiation of autophagy, as evidenced by the slightly up-regulated expression of Beclin1, Vps34 and LC3B-II; however, these effects were markedly enhanced by Nrf2 knockout. Increasing studies have reported that mitochondrial dysfunction is associated with glucose metabolism. For instance, the impaired insulin-induced abnormal glucose metabolism was attributed to mitochondrial fission in skeletal muscle [56]. Our previous studies confirmed that PM_2.5_ exposure led to insulin resistance in liver tissues, contributing to non-alcoholic fatty liver disease with significantly increased blood glucose levels [17]. Here, we found that PM_2.5_ treatment led to abnormal glucose metabolism in cardiac samples, as evidenced by the decreased expression of glycolytic enzymes, such as GCK, PK and HK1, along with the increased gluconeogenic enzymes G6Pase and PEPCK [37–40]. Of note, these effects were markedly aggravated when Nrf2 was deleted. Numerous studies have implied the essential of Nrf2 in maintaining glycometabolism partly associated with the mitochondrial function [57, 58]. Therefore, we hypothesized that PM_2.5_-induced cardiac injury was related to the degradation of glucose metabolism regulated by Nrf2. Furthermore, promoting Nrf2 expression by DMF could alleviate PM_2.5_-induced cardiomyopathy in the wild type mice. It has been suggested that genetic impairments of Nrf2 could result in metabolic disturbance in cardiovascular systems under stress conditions [59]. Thereby, our results also supported the notion that prolonged PM_2.5_ exposure elevates adverse influences to individuals with underlying cardiometabolic diseases.

The adverse effects of PM_2.5_ exposure on cardiovascular diseases and pulmonary system are well indicated [2, 3, 49, 50]. These harmful effects triggered by PM_2.5_ might be involved in the oxidative stress mechanism, which is also known to play an essential role in the pathogenesis of cardiac fibrosis and inflammation [60, 61]. Thus, PM_2.5_ exposure might be associated with collagen accumulation and inflammatory response in hearts through the oxidative stress mechanism. Nrf2 plays a critical role in the improvement of various inflammatory- and oxidative stress-induced diseases [62, 63]. Our study suggested that exposure to the high level of real-world ambient PM_2.5_ for 6 months significantly increased fibrosis in hearts of Nrf2^+/+^ mice, as evidenced by the up-regulation of Col1a1, α-SMA, FN and TGFβ1. Col1a1 is one of the major collagen proteins of the extracellular matrix in tissues. FN and α-SMA are characterized by fibrogenesis [64]. TGFβ1 is central to the pro-fibrotic switch, activation of myofibroblasts and matrix accumulation [65]. Previous studies also showed that PM_2.5_ exposure alone could cause mild hepatic fibrosis and activation of the TGFβ-Smad3 signaling pathway [66, 67]. Consistently, our western blot analysis suggested that PM_2.5_ exposure led to a significant increase of TGFβ1, p-Smad2 and p-Smad3 in heart tissues. On the other, overproduction of pro-inflammatory cytokines regulated by IκBα/NF-κB signaling also contributes to the cytotoxicity of PM_2.5_ [15, 17, 68, 69]. Thereby, the increased levels of serum or heart TNFα, IL-1β and IL-6, and the up-regulated expression of p-IκBα and p-NF-κB in hearts from PM_2.5_-exposed Nrf2^+/+^ mice provided an alternate essential mechanism for the cardiac dysfunction observed herein. Interestingly, PM_2.5_-induced fibrosis and inflammatory response were further accelerated by the Nrf2^-/-^.

Transcription factor Nrf2 is a master regulator of numerous cytoprotective genes, which has been a potential target for the treatment of a variety of cardiovascular diseases [10, 11]. Nrf2 is anchored in the cytoplasm where it binds to Keap1 under normal condition. However, Nrf2 could translocate into the nucleus and then activate its target genes through an antioxidant-response element (ARE). Nrf2 thus can negatively modulate the dissociation and polymerization with Keap1, subsequently adjust the expression of some anti-oxidative genes or enzymes against oxidative stress, such as SOD, HO1, NQO1, GSH and GCLM [9, 12–14]. In our study, we found that PM_2.5_ exposure caused significant oxidative stress in heart tissues, as evidenced by the reduction of SOD, HO1, NQO1, GSH and GCLM. In contrast, subsequent products or hallmarks for oxidative stress, including MDA, 4-HNE, 3’-NT, 8-OHdG, NOX2 and NOX4 [16, 70, 71], were found to be significantly up-regulated by PM_2.5_ exposure. As expected, Nrf2 knockout further accelerated PM_2.5_-triggered oxidative stress in cardiac samples, which contributed to the collagen accumulation and inflammatory response. In addition, our *in vitro* experiments confirmed the protective role of Nrf2 against PM_2.5_-induced ROS generation, fibrosis and inflammation. Importantly, we found that preventing ROS production by NAC or elevating HO1 activation by CoPPIX could significantly alleviate Nrf2^-/-^-promoted oxidative stress, fibrosis and inflammation in cardiomyocyte stimulated by PM_2.5_. Therefore, Nrf2-regulated oxidative stress played a critical role in PM_2.5_-induced cardiac injury through modulating fibrosis and inflammation.

Our previous study showed that RIPK3-mediated oxidative stress and inflammation were involved in metabolic stress-induced hepatic injury associated with Nrf2 signaling [26]. Recently, RIPK3 was suggested to promote necroptosis in cardiac ischemia-reperfusion injury through activating ROS production [25]. A strong evidence reported that RIPK3-dependent mitochondrial dysfunction led to compromised kidney tubular epithelial cell function, contributing to acute kidney injury [27]. RIPK3-dependent signaling results in mitochondrial depolarization and decreases expression of mitochondrial complex subunits in response to endotoxin. Genetic RIPK3 knockout preserves ATP production in cells following lipopolysaccharides stimulation [27]. Furthermore, accumulating evidence demonstrate that changes in mitochondrial dynamics such as structural and functional aberrations in mitochondria have been implicated in cardiovascular diseases, including cardiomyocyte hypertrophy and ischemia-reperfusion injury [72, 73]. Mitochondria could reach a kind of equilibrium between mitochondrial fusion and fission, which are essential for mitochondrial growth, redistribution and maintenance of a healthy mitochondrial function. A significant mechanism of ambient PM_2.5_ exposure-induced acute heart injury in rats was attributed to mitochondrial damage [74]. In our study, we also found that PM_2.5_ exposure led to a significant expression of RIPK3 in heart tissues, attendant with reduced ATP production and mitochondrial Complex I respiration, while increased mtDNA levels. In addition, mitochondrial fusion is regulated by two mitofusins (MFN1 and MFN2) in outer membrane and Opa1 in inner membrane [75]. Fis1 is also involved in mitochondrial fission through recruiting the cytoplasmic Drp1 into the mitochondrial outer-membrane. Drp1 can punctuate spots on the mitochondrial surface to regulate mitochondrial fission for meeting energy needs [76]. MID49 and MID51, as receptors for the mitochondrial fission protein Drp1, are also crucial mediators of mitochondrial fission and novel targets for cardioprotection [77]. Here, our study also demonstrated that PM_2.5_ exposure led to significant up-regulation of Fis1, Drp1, Mid51 and Mid49, and down-regulation of MFN1, MFN2 and Opa1 in heart tissues. These results indicated that long-term PM_2.5_ exposure caused mitochondrial injury and dysfunction, which was involved in cardiac injury. As reported, Nrf2 knockout exacerbates frailty and sarcopenia through impairing skeletal muscle mitochondrial biogenesis and dynamics [78]. The function of Nrf2 is inhibited in mitochondria-associated disorders. Nrf2 deletion results in impaired mitochondrial respiration and ATP production [79]. In the present work, we demonstrated that PM_2.5_-induced increase of RIPK3 and mitochondrial disorder was further accelerated in Nrf2^-/-^ mice. The *in vitro* experiments suggested that blocking RIPK3 expression could attenuate Nrf2 deletion-enhanced mitochondrial disorder in PM_2.5_-incubated cardiomyocytes, as evidenced by the rescued mitochondrial potential, ATP production and the reduced mPTP opening. As a consequence, mPTP opening causes mitochondrial potential collapse, oxidative phosphorylation attest and ATP undersupply [80]. We also found that the function of mitochondrial complex I-IV, as proved by the expression of NDUFB8, SDHB, UQCRC1 and MTCO1, respectively [41, 42], were declined in PM_2.5_-stimulated cardiomyocytes in a manner through RIPK3. Thus, Nrf2-regulated mitochondrial dysfunction induced by PM_2.5_ was dependent on RIPK3 expression, which further contributed to ROS overproduction, fibrosis and inflammation. However, the role of other mitophagy regulators in PM_2.5_-induced cardiovascular disease requires further investigation.

Previous studies indicated that Nrf2 was significant for the protection against multiple stresses-induced cardiovascular diseases [81, 82]. In the present study, Nrf2 was found to be involved in the protection of PM_2.5_-induced cardiac injury by regulating oxidative stress, fibrosis and inflammation via RIPK3-meditated mitochondrial disorder. To confirm our hypothesis, Nrf2 activator of DMF, a drug approved for treatment of multiple sclerosis and psoriasis by activating Nrf2-responsive genes [83], was then subjected to mice with PM_2.5_ exposure. We found that DMF exhibited beneficial effects in PM_2.5_-induced cardiomyopathy by inhibiting RIPK3 expression, oxidative stress, fibrosis and inflammation via the up-regulation of Nrf2.

**Figure 10:**
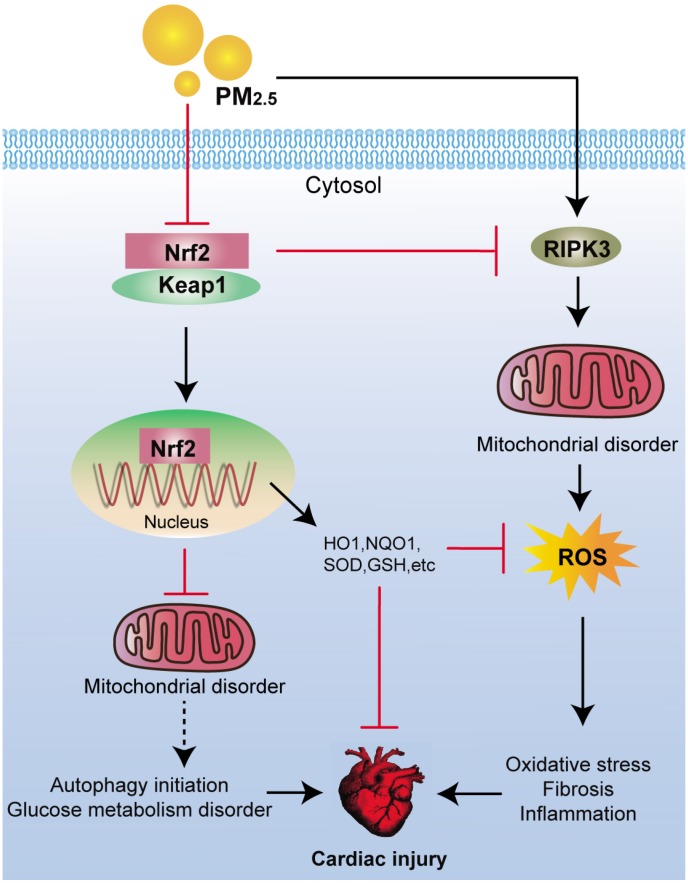
**Scheme of the Nrf2-mediated cardiac injury induced by PM_2.5_.** Long-term exposure of PM_2.5_ to mice resulted in oxidative stress, fibrosis and inflammation via RIPK3-regulated mitochondrial disorder. In addition, autophagy initiation and dysfunction of glucose metabolism were observed in hearts of PM_2.5_-challenged mice. All these effects induced by PM_2.5_ were significantly accelerated by the loss of Nrf2, contributing to the progression of cardiomyopathy eventually.

In conclusion, our present study indicated that Nrf2 could protect against PM_2.5_ exposure-induced cardiac injury through suppressing RIPK3-regulated mitochondrial dysfunction, thereby inhibiting ROS production, collagen accumulation and inflammatory response. Moreover, PM_2.5_ exposure-induced oxidative stress was partly attributed to glucose metabolism disorder regulated by Nrf2, contributing to cardiac injury (Figure 10). All these finding may highlight that improving Nrf2 activity is a promising strategy to treat air pollution-induced cardiovascular disease.

## MATERIALS AND METHODS

### PM_2.5_ sampling preparation

The method for PM2.5 sampling preparation is in accordance with Ying et al. and Ogino et al. [84, 85] procedures with certain modification. In brief, to collect exposure mass, quartz filter (8 cm × 10 cm, 2500QAT-UP, Pallflex Products, Putnam, CT, USA) was used to continuously and weekly gather PM_2.5_ from Yuquan Road, Beijing, China (January-June 2015) at a flow rate of 166 L/min. Ambient PM_2.5_ filters were then stored in -80°C until administration. The sampling was treated with anhydrous alcohol and dissolved in pyrogen-free water. Next, the extraction was sonicated for 48 h in ultrasonic box and then concentrated by vacuum freeze-drying. Double-distilled water was then added to freeze-dried product, which was subsequently centrifuged at 5000 rpm. The water-insoluble fraction was suspended in D-Hank’s buffer (GIBCO Corporation, Gaithers- burg, MD, USA) and vortexed prior to further analysis. The components of PM_2.5_ were listed in Supplementary Table 1.

### Ethical approval and animals experimental design

The male, 6-8 weeks of age, weighed 20 ± 2 g, wild type C57BL/6J mice (Nrf2^+/+^) and Nrf2 knockout C57BL/6J mice (Nrf2^-/-^) were purchased from the Jackson Laboratory (Bar Harbor, ME). Prior to the animal experiments, all mice were subjected to adapt to the environment for 1 week. They were housed in a specific pathogen-free (SPF), temperature- and humidity-controlled environment (25 ± 2°C, 50 ± 5% humidity) with a standard light/dark (12h/12h) cycle and free access to food and water in their cages. All procedures were in accordance with the Care and Use of Laboratory Animals, which was published by the US National Institutes of Health (NIH Publication No. 85-23, revised 1996). The Institutional Animal Care and Use Committee at Chongqing Key Laboratory of Medicinal Resources in the Three Gorges Reservoir Region, School of Biological and Chemical Engineering, Chongqing University of Education (81703527) approved the animal study protocols. After one-week acclimatization, all mice were randomly divided into 5 groups: 1) Nrf2^+/+^/FA group; 2) Nrf2^-/-^/FA group; 3) Nrf2^+/+^/PM_2.5_ group; 4) Nrf2^-/-^/PM_2.5_ group and 5) Nrf2^+/+^/PM_2.5_+DMF group. Mice were exposed to concentrated PM_2.5_ (150.1 ± 2.5 μg/m^3^, flow rate of 65 L/min) or filtered air (FA, served as control) for 6 h/day, 5 times a week in a mobile exposure system-HOPE-MED 8052 automatic nose and mouth type inhalation exposure system (Hepu Industry and Trade Co., Ltd., China) according to previous studies [15, 16, 86, 87]. Nrf2^+/+^ mice were received intraperitoneal injections of dimethyl fumarate (DMF, 25 mg/kg/day) for 6 h, and then treated with PM_2.5_. After PM_2.5_ exposure for 24 weeks with or without DMF, all mice were sacrificed for blood collection. Body weight of mice was recorded every week. Blood pressure was measured each week by a noninvasive blood pressure meter (Surgivet, USA). The lung and heart tissue samples were isolated from mice for further analysis. DMF was purchased from Sigma-Aldrich (St. Louis, MO, USA) for animal treatments.

### Cells isolation and culture

Mouse cardiomyocytes were isolated from the Nrf2^+/+^ or Nrf2^-/-^ mice as previously indicated [88, 89]. In brief, hearts from mice were mounted onto a temperature-controlled (37°C) system. After perfusion with a modified Tyrode’s solution (Sigma Aldrich), the heart was digested using a Ca^2+^-free Krebs Henseleit-bicarbonate (KHB) buffer supplemented with liberase blendzyme 4 (Roche, Norway) for 20 min. The modified Tyrode solution was gassed with 5% CO_2_. Then, the digested heart was removed from the cannula and the left ventricle was cut into small pieces in the modified Tyrode’s solution. Heart tissue pieces were gently agitated, and then pellet of cells was resuspended. A yield of 65% viable rod-shaped cardiomyocytes with clear sacromere striations was acquired. Only rod-shaped myocytes with clear edges were chose for *in vitro* studies. Control and RIPK3-specific siRNAs were synthesized and purchased from Shanghai Generay Biotech (Shanghai, China) and transfected into cells using Lipofectamine® 3000 (Invitrogen, USA) following the manufacturer’s protocol. HO1 activator of CoPPIX, and ROS scavenger of NAC were purchased from Sigma-Aldrich. RIPK3 inhibitor of GSK872 (#HY-101872) was purchased from MedChemExpress (USA) to suppress RIPK3 expression.

### Biochemical analysis

Blood glucose levels were calculated by glucometer (5D-1, ACCU-CHEK, China) every month. The concentration of CK, LDH, MDA, SOD, GSH-Px, GSH and iNOS in fresh serum and heart tissues immediately extracted from each group of mice, or cells were measured using the commercial kits (Nanjing Jiancheng Bioengineering Institute, Nanjing, China) according to the manufacturer’s instructions. Glucokinase (GCK), pyruvate kinase (PK) and succinate dehydrogenase (SDH) activities in hearts of mice were measured using the Assay Kit also purchased from Nanjing Jiancheng Bioengineering Institute according to the instruction recommended by the manufacturer. The levels of 4-HNE in hearts were measured using mouse 4-HNE Elisa kit (Shanghai Shuangying Biotech, Shanghai, China) according to the manufacturer’s instructions. The 3’-NT and 8-OHdG in cardiac samples were measured using commercial kits purchased from Abcam (USA) strictly according to the manufacturer’s instructions. Serum levels of tumor necrosis factor-α (TNF-α, #MTA00B), interleukin 1β (IL-1β, #MLB00C) and IL-6 (#M6000B) were measured using enzyme-linked immunosorbent assay (ELISA) commercial kits (R&D System, USA) according to the manufacturer’s instructions.

### BALF isolation and calculation

After 6 months of exposure, the mice were anesthetized using pentobarbital sodium (20 mg/kg, iv). Then, BALF was collected by lavaging lungs with 1.0 mL of sterile PBS. The BALF was centrifuged at 1000 rpm for 10 min and then suspended in 100 μl of PBS to count the total cell and calculate protein concentration with a Bicinchoninic acid (BCA) protein analysis kit (Thermo Fisher Scientific, USA) according to the manufacturer’s protocol.

### Echocardiography

At the end of animal experiments, all mice were anesthetized using 2,2,2-Tribromethanol (TBE, 200 mg/kg, MedChemExpress, USA). Cardiac function was then assessed through ECG using an Acuson Sequoia C256 System (Siemens) ultrasound machine. For each animal, LVFS%, LVEF%, LVIDd and LVIDs were measured as previously indicated [90, 91].

### Isolation of cytosolic and mitochondrial fractions

Cytosolic and mitochondrial fractions were isolated from cells by a mitochondria/cytosol fraction kit (Biovision, USA) according to the manufacturer’s protocols. The cytosolic glyceraldehyde-3-phosphate dehydrogenase (GAPDH) and mitochondrial inner membrane protein TIM23 were served as standards for cytosol and mitochondrial protein, respectively [92].

### Western blots and quantitative real-time PCR (RT-qPCR) analysis

For western blot analysis, heart tissues and cells were homogenized using 10% (wt/vol) hypotonic buffer (pH 8.0) to yield a homogenate. Bicinchonic acid (BCA) protein analysis kit (Thermo Fisher Scientific) was used to determine the protein concentration according to its instructions. 20-40 μg protein from hearts, cells or mitochondrial fractions was subjected to 10% SDS-Polyacrylamide-Gel-Electrophoresis (SDS-PAGE) and transferred to polyvinyldene fluoride (PVDF) membranes (Millipore, USA), followed by incubation with primary antibodies (Supplementary Table 2) overnight at 4°C. Then, the membranes were incubated with corresponding secondary antibodies (Supplementary Table 2) for 1 h at room temperature. The signal was detected using enhanced chemiluminescence (ECL) Detection system (Thermo Fisher Scientific). Each protein expression was analyzed using Image Lab Software (Version 1.4.2b, National Institutes of Health, USA), normalized to GAPDH and expressed as a fold of control.

As for RT-qPCR, total RNA from cardiac tissue samples or cells was extracted using Trizol reagent (Invitrogen, USA) according to the manufacturer’s introductions. Total RNA was reverse transcribed using M-MLV-RT system (Promega, USA). The action was performed at 42°C for 1 h and terminated via deactivation of the enzyme at 70°C for 10 min. Then, PCR was performed by SYBR Green (Bio-Rad, USA) on an ABI PRISM 7900HT detection system (Applied Biosystems, USA). All primer sequences included in the present study were purchased from Invitrogen Corporation or Generay Biotech (Shanghai, China) and listed in Supplementary Table 3. The quantification analysis was performed according to the 2^-ΔΔCt^ methods [93]. Target RNA levels were normalized to GAPDH.

### Mitochondrial DNA (mtDNA) determination

Total DNA was extracted from myocardium using phenol/chloroform/isoamyl alcohol (25:24:1), which was followed by isopropanol precipitation. The mtDNA levels were determined through calculating mitochondrial gene cytochrome oxidase subunit 1 (Cox1) (forward 5’-ACT ATA CTA CTA CTA ACA GAC CG-3’, reverse 5’-GGT TCT TTT TTT CCG GAG TA-3’) against nuclear gene cyclophilin A (forward 5’-ACA CGC CAT AAT GGC ACT GG-3’, reverse 5’-CAG TCT TGG CAG TGC AGA T-3’) by RT-qPCR via the comparative 2^−ΔΔCt^ method.

### Histological analysis

The heart and lung samples extracted from mice were fixed with 4% paraformaldehyde, implanted in paraffin, and sectioned transversely. Sections at 3 μm thickness were subjected to hematoxylin followed by eosin (H&E) staining, Masson’s trichrome staining and Sirius Red staining for the tissues injury and collagen accumulation calculation [94] derived from 3 pathologists under a light microscopy (Olympus, Tokyo, Japan).

### IHC analysis

For immunostaining of 8-OHdG (ab48508, Abcam), p-NF-κB (ab28856, Abcam) and RIPK3 (ab56164, Abcam), cardiac tissues were incubated in 3% H_2_O_2_ for 10 min to block endogenous peroxidase activity. Then, 5% bovine serum albumin (BSA, Shanghai Boao Biotechnology Co., Ltd., Shanghai, China) was used to block non-specific binding for 1 h. Then, heart tissues were incubated with primary antibodies (dilutions at 1:200) overnight at 4°C, followed by incubation with secondary antibodies at 37°C for 30 min. After washing with PBS, the sections were developed using 3,3’-diaminobenzidine (DAB, Sigma Aldrich) with 0.03% hydrogen peroxide for 5 min. Then, tissue sections were counterstained with hematoxylin for 1 min and observed under a light microscope (Olympus).

### Mitochondrial function and respiration measurement

Mitochondrial function was performed through calculating ATP production and mitochondrial potential [95]. The JC-1 Kit (Beyotime, Nanjing, China) was used to measure the change in the mitochondrial membrane potential [96]. The cellular ATP levels were measured using a firefly luciferase-based ATP assay kit (Beyotime) based on a fluorescence technique according to the manufacturer’s instructions. As for ATP calculation in heart tissues, ATP Assay Kit (ab83355, Abcam) used the phosphorylation of glycerol to generate a product quantified by colorimetric method. 10 mg of heart tissue was homogenized in 100 μl of ATP assay buffer and centrifuged. The supernatant was then transferred for deproteinization using ice-cold 4 M perchloric acid and 2 M KOH. The analysis was performed according to the manufacturer’s instruction. The mPTP opening was measured through calcein-AM/cobalt. The relative mPTP opening rate was measured as the ratio to control group [97]. As for mitochondrial respiration measurement, hearts were cut into longitudinal strips along fiber orientation with a diameter of 1.5 mm. Then, the fiber bundles were transferred to a tube with 2 ml ice-cold relaxation and preservation solution with 50 μg/ml saponin (Sigma-Aldrich) for 20 min permeabilization [98]. Fibers were rinsed with fresh mitochondrial respirometry solution MiR05 (0.5 mM EGTA, 3 mM MgCl_2_, 60 mM K-lactobionate, 10 mM KH_2_PO_4_, 20 mM HEPES, 110 mM sucrose, pH 7.1). 2 mg cardiac fiber was placed in the oxygraph chambers supplemented with MiR05 followed by addition of 10 mM glutamate and 5 mM malate for evaluating complex I-supported Leak respiration rate. Then, 2 mM ADP was added to determine the maximal phosphorylating respiration rate.

### Cellular viability and intracellular ROS

The 3-(4,5- dimethylthiazol-2-y1)-2,5-diphenyl tetrazolium bromide (MTT) assay was performed to analyze the cell viability according to the manufacturer’s instructions (Beyotime). Absorbance was determined at 570 nm. The relative cell viability was recorded as a ratio to that in the control group. After various treatments, 1.5 mL of dichloro-dihydro-fluorescein diacetate (DCFH-DA) (10.0 μM, Sigma-Aldrich) was added at 37°C for 25 min, and then the samples were analyzed using a fluorescence microscopy (Olympus, Tokyo, Japan).

### Statistical analysis

All data in this study are expressed as the mean ± standard error of the mean (SEM). For comparison between two groups, Student’s two-tailed t-test was used. For comparisons among multiple groups, parametric statistical analysis was conducted using one-way analysis of variance (ANOVA) with Tukey post hoc test. A P value < 0.05 was considered statistically significant. Statistical analysis was performed using GraphPad PRISM (version 6.0 for mac; Graph Pad Software).

## Supplementary Material

Supplementary Figure 1

Supplementary Tables
