# Supplementary material for: Nrf2 deficiency aggravates PM2.5-induced cardiomyopathy by enhancing oxidative stress, fibrosis and inflammation via RIPK3-regulated mitochondrial disorder
Source: Aging (Albany NY). 2020 Mar 17;12(6):4836–65. doi: 10.18632/aging.102906 (PMC7138545; doi:10.18632/aging.102906)
Supplement: Supplementary Figure 1 [file aging-12-102906-s001..pdf]

SUPPLEMENTARY FIGURE

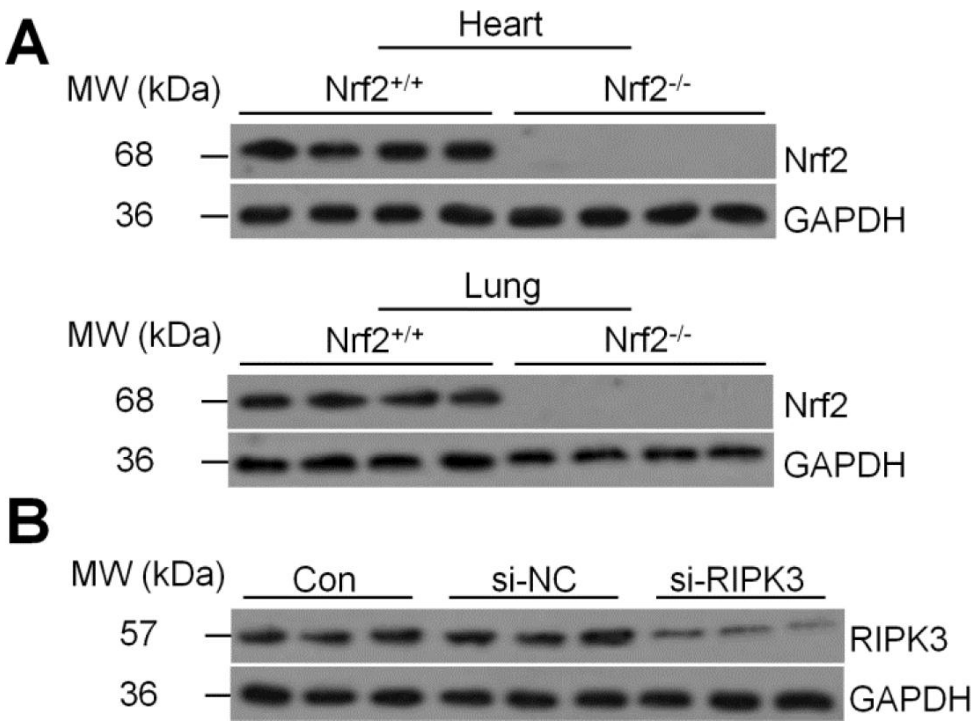

Supplementary Figure 1. (**A**) Western blot analysis of Nrf2 in lung (up panel) and heart (down panel) tissues from the Nrf2$^{+/+}$ mice or Nrf2$^{-/-}$ mice (n=8/group). (**B**) Cardiomyocytes were transfected with siRIPK3 or its corresponding negative control siRNA for 24 h. Then, the cells were collected for western blot analysis of RIPK3 (n=6/group).
