# Supplementary material for: Nrf2 deficiency aggravates PM2.5-induced cardiomyopathy by enhancing oxidative stress, fibrosis and inflammation via RIPK3-regulated mitochondrial disorder
Source: Aging (Albany NY). 2020 Mar 17;12(6):4836–65. doi: 10.18632/aging.102906 (PMC7138545; doi:10.18632/aging.102906)
Supplement: Supplementary Tables [file aging-12-102906-s002..pdf]

SUPPLEMENTARY TABLES

Supplementary Table 1. Components of $PM_{2.5}$ (ng/m^3).

Element	Ambient $PM_{2.5}$
Zinc	11.19 ± 3.71
Arsenic	0.96 ± 0.14
Lanthanum	0.02 ± 0.01
Barium	3.02 ± 0.68
Antimony	0.55 ± 0.24
Molybdenum	0.45 ± 0.23
Titanium	0.83 ± 0.25
Calcium	88.46 ± 12.51
Sulfur	1021.28 ± 346.71
Phosphorus	17.21 ± 2.55
Aluminum	23.44 ± 7.13
Magnesium	26.75 ± 3.96
Sodium	55.19 ± 11.47
Lead	2.91 ± 0.74
Copper	2.45 ± 0.74
Potassium	37.23 ± 8.87
Selenium	0.46 ±0.04
Cerium	0.04 ± 0.01
Cadmium	0.51 ± 0.06
Strontium	0.36 ± 0.11
Rubidium	0.06 ± 0.01
Nitrogen	0.43 ± 0.51
Cobalt	0.12 ±0.11
Iron	55.63 ± 13.96
Manganese	1.88 ± 0.63
Chromium	8.47 ± 1.15
Vanadium	0.31 ± 0.19

Supplementary Table 2. Primary and secondary antibodies used for western blot analysis.

Name	Catalog	Dilutions	Vendor
TGFβ1	ab93650	1:1000	Abcam
Phospho-NF-κB	ab86299	1:1000	Abcam
NF-κB	ab76311	1:1000	Abcam
IκBα	#4814	1:1000	Cell Signaling Technology
Phospho-IκBα	#2859	1:1000	Cell Signaling Technology
RIPK3	ab56164	1:1000	Abcam
HO1	ab13248	1:1000	Abcam
Nrf2	ab31163	1:1000	Abcam
GCLM	ab126704	1:1000	Abcam
α-SMA	ab32575	1:1000	Abcam
NDUFB8	ab110242	1:1000	Abcam
SDHB	ab14714	1:1000	Abcam
UQCRC1	#459140	1:1000	Invitrogen
MTCO1	ab14705	1:1000	Abcam
TIM23	ab230253	1:1000	Abcam
Phospho-Smad-2	#18338	1:1000	Abcam
Smad-2	#5339	1:1000	Abcam
Keap-1	ab150654	1:1000	Abcam
Phospho-Smad-3	#9523	1:1000	Cell Signaling Technology
Smad-3	#9520	1:1000	Cell Signaling Technology
Beclin1	ab210498	1:1000	Abcam
Vps34	ab124905	1:1000	Abcam
LC3B	ab192890	1:1000	Abcam
ATG5	ab108327	1:1000	Abcam
Rabbit IgG	ab6721	1:4000	Abcam
Mouse IgG	ab6728	1:4000	Abcam
GAPDH	ab8245	1:1000	Abcam

Supplementary Table 3. Primer sequences for RT-qPCR analysis.

Gene name	Primer sequence (5→3′)
ANP (Mus musculus)	Forward primer ACCTGCTAGACCACCTGGAG
	Reverse primer CCTTGGCTGTTATCTTCGGTACCGG
BNP (Mus musculus)	Forward primer GAGGTCACTCCTATCCTCTGG
	Reverse primer GCCATTTCCTCCGACTTTTCTC
Col1a1 (Mus musculus)	Forward primer AGGCTTCAGTGGTTTGGATG
	Reverse primer CACCAACAGCACCATCGTTA
α-SMA (Mus musculus)	Forward primer TTCCTTCGTGACTACTGCCG
	Reverse primer TATAGGTGGTTTCGTGGATGCC
FN (Mus musculus)	Forward primer CACCCGTGAAGAATGAAG
	Reverse primer CAGGCAGGAGATTTGTTAG
TGF-β1 (Mus musculus)	Forward primer TGGCCAGATCCTGTCCAAAC
	Reverse primer CATAGATGGCGTTGTTGCGG
SOD1 (Mus musculus)	Forward primer GAGACCTGGGCAATGTGACT
	Reverse primer GTTTACTGCGCAATCCCAAT
SOD2 (Mus musculus)	Forward primer ATTAACGCGCAGATCATGCA
	Reverse primer TGTCCCCCACCATTGAACTT
NOX2 (Mus musculus)	Forward primer TGTGGCTGTGATAAGCAGGA
	Reverse primer TCCCACTAACATCACCACCT
NOX4 (Mus musculus)	Forward primer GATTTCTGGACCTTTGTGCCTTT
	Reverse primer TGATGGTGACAGGTTTGTTGCT
TNF-α (Mus musculus)	Forward primer ACCTGGCCTCTCTACCTTGT
	Reverse primer CCCGTAGGGCGATTACAGTC
IL-1β (Mus musculus)	Forward primer GCCACCTTTTGACAGTGATGAG
	Reverse primer AGTGATACTGCCTGCCTGAAG
RIPK3 (Mus musculus)	Forward primer ATGTCTAAACTCTCAGCCGTA
	Reverse primer ATTGAGCCATAACTTGACAGA
Fis1 (Mus musculus)	Forward primer GTAGGGTTACATGGATGCCCAGAGA
	Reverse primer GGCAAAAGCTCCTCCAGCAG
Drp1 (Mus musculus)	Forward primer CGGTTCCCTAAACTTCACGA
	Reverse primer GCACCATTTCATTTGTCACG
Mid51 (Mus musculus)	Forward primer AGGATGACAATGGCATTGGC

	Reverse primer CCGATCGTACATCCGCTTAAC
Mid49 (Mus musculus)	Forward primer GCAACCAATCCACCAACAGAAT<br>Reverse primer CCGGAAAAGGCGTTAAGTCAC
MFN1 (Mus musculus)	Forward primer CCATCACTGCGATCTTCGGCCA<br>Reverse primer CAGCGAGCTTGTTTCTGTAGCCCT
MFN2 (Mus musculus)	Forward primer GGGGCCTACATCCAAGAGAG<br>Reverse primer GCAGAACTTTGTCCCAGAGC
Opa1 (Mus musculus)	Forward primer GATGACACGCTCTCCAGTGAAG<br>Reverse primer CTCGGGGCTAACAGTACAACC
GCK (Mus musculus)	Forward primer TATGAAGACCGCCAATGT<br>Reverse primer CCGCCAATGATCTTTTC
PK (Mus musculus)	Forward primer TTCTGTCTCGCTACCGA<br>Reverse primer GTCACCACAATCACCAG
HK1 (Mus musculus)	Forward primer TGCCAAGCTAACTCCAAACC<br>Reverse primer GATACAGTCCGCTTCGGGTA
G6Pase (Mus musculus)	Forward primer ACTGTGGGCATCAATCTCCTC<br>Reverse primer CGGGACAGACAGACGTTCAGC
PEPCK (Mus musculus)	Forward primer CTGCATAACGGTCTGGACTTC<br>Reverse primer GCCTTCCACGAACTTCCTCAC
GAPDH (Mus musculus)	Forward primer GGTGAAGGTCGGTGTGAACG<br>Reverse primer CCCGTAGGGCGATTACAGTC
